# Intestinal Viral Loads and Inactivation Kinetics of Livestock Viruses Relevant for Natural Casing Production: A Systematic Review and Meta-Analysis

**DOI:** 10.3390/pathogens10020173

**Published:** 2021-02-04

**Authors:** Tinka Jelsma, Joris J. Wijnker, Wim H. M. van der Poel, Henk J. Wisselink

**Affiliations:** 1 Department of Virology, Wageningen Bioveterinary Research (WBVR) Part of Wageningen University & Research (WUR), P.O. Box 65, 8200 AB Lelystad, The Netherlands; wim.vanderpoel@wur.nl; 2 Department of Population Health Sciences, Institute for Risk Assessment Sciences, Faculty of Veterinary Medicine, Utrecht University, P.O. Box 80178, 3508 TD Utrecht, The Netherlands; j.j.wijnker@uu.nl; 3 Department of Infection Biology, Wageningen Bioveterinary Research (WBVR) Part of Wageningen University & Research (WUR), P.O. Box 65, 8200 AB Lelystad, The Netherlands; henk.wisselink@wur.nl

**Keywords:** natural casings, intestines, animal viruses, viral loads, titers, inactivation, D-value

## Abstract

Animal intestines are the source of edible sausage casings, which are traded worldwide and may come from areas where notifiable infectious animal diseases are prevalent. To estimate the risks of virus contamination, knowledge about the quantity of virus and decimal reduction values of the standard preservation method by salting is of great importance. A literature search, based on the Preferred Reporting Items for Systematic Reviews and Meta-Analyses (PRISMA) guidelines, was performed in search engine CAB Abstracts to determine the viral load of 14 relevant animal viruses in natural casings or intestines. Only a very limited number of scientific publications per virus were found and viral loads in the intestines varied from high for ASFV (five publications), BVDV (3), CSFV (6), PPRV (3), RPV (2) and TGEV (3) to moderate for PEDV (2) and SVDV (3), low for HEV (2) and FMDV (5), very low for VESV (1) and negative for PrV (2) and VSV (1). PRRSV was found in intestines, however, viral titers were not published. Three viruses (BVDV, CSFV and PPRV) with high viral loads were selected to search for their inactivation kinetics. For casings, no inactivation data were found, however, thermal inactivation data of these viruses were available, but differed in quantity, quality and matrices. In conclusion, important data gaps still exist when it comes to the quantitative inactivation of viruses in sausage casings or livestock intestines.

## 1. Introduction

### 1.1. Natural Casings

The natural casing is the edible container of sausages and is mainly derived from the small or large intestine of either pig, sheep, goat or cattle. For example, to produce a natural casing from the small intestines of pigs and sheep, the intestines are flushed with water and scraped for the removal of the inner and outer layers of the intestinal wall. The remaining submucosa layer will form, after cleaning with water and incubation with saturated salt for preservation, the natural casing. Because natural casings are being produced as well as traded worldwide, contagious animal viruses from all around the world may be present in this product. For this reason, international trade is subject to animal and public health guidelines. In order to estimate the risks of contamination, knowledge about the quantity of virus and the decimal reduction values (D-values) obtained by the inactivation process is of great importance. The aim of this systematic review (SR) is to gather information about viral loads present in the intestines of the animal host. Additionally, a literature search concerning the inactivation kinetics of three animal viruses found with high viral loads was performed and if possible D-values were calculated and were meta-analyzed. Overall, the results should provide more insight into the knowledge gaps (viral loads and virus inactivation in intestines), and subsequently give an indication of which viruses are of major concern for the natural casing industry.

### 1.2. Animal Viruses Selection

Fourteen animal viruses were selected based on their worldwide relevance and impact on the food and livestock industry. Nine viruses are listed by the OIE (World Organization for Animal Health), as notifiable diseases, these include African swine fever virus (ASFV), classical swine fever virus (CSFV), Foot and mouth disease virus (FMDV), Peste des petits ruminants virus (PPRV), Porcine reproductive and respiratory syndrome virus (PRRSV), Pseudorabies virus (PrV), Rinderpest virus (RPV), Transmissible gastroenteritis virus (TGEV), Bovine viral diarrhea virus (BVDV) [1]. Besides OIE-listed viruses, Hepatitis E virus (HEV), Porcine epidemic diarrhea virus (PEDV), Swine vesicular disease virus (SVDV), Vesicular exanthema of swine virus (VESV) and Vesicular stomatitis virus (VSV) were described in risk assessment reports and in a scientific EFSA opinion relevant for the natural casing industry [2,3,4].

## 2. Results

### 2.1. Q1 Viral Load

The search concerning the viral loads of 14 viruses in the intestines resulted in a very limited number of publications per virus as indicated in Table 1 (right column).

#### 2.1.1. African Swine Fever Virus (ASFV) Characteristics

African swine fever virus (ASFV) is a large enveloped double-stranded DNA virus of the genus Asfivirus within the Asfarviridae family [5]. African swine fever (ASF) is one of the most important viral OIE-listed diseases of domestic pigs, resulting in major impacts on animal health and pig production industry and was first described from Kenia [1,6]. Recently, outbreaks occurred outside Africa, in South and Eastern Europe and Asia. China was the first country in Asia to be affected by ASF and soon other countries followed including almost all of South-East Asia and also spreading to Indonesia, the Philippines and Papua Guinea [7]. The main course seems to be swill feeding pigs with contaminated pork meat/meat products in small farms with less than 50 pigs (backyard farming), however, the importance of this transmission route was questioned in a recent publication, concluding that the most important transmission route of ASFV over long distances is mainly caused by human behavior [8]. No vaccine is available against ASF and current disease control is based on stamping out procedures. 

#### 2.1.2. ASFV Intestinal Viral Load

ASFV was found present in intestines in relative high titers, up to 6.2 log_10_ HAD_50_/g in the ileum at 6 days post-inoculation (dpi). Blood titers on the same day were 8.2 log_10_ HAD_50_/mL [9]. Infectious ASFV survived in processed salted casings for 97 days, with an initial titer of 3.5 log_10_ HAD_50_/g [10]. These titers were confirmed in a recent study, showing a maximum virus load of 5.3 log_10_ TCID_50_-eq/g in the ileum at 7 dpi. Furthermore, high titers were found throughout the entire intestine [11]. Because the amount of virus found in the blood was higher than in the intestines, it is most likely that the viremia contributes to the height of the intestinal viral load [9,11,12]. A link found recently between increasing virus inoculation doses and increasing colon titers (genome copy numbers/g) may confirm this hypothesis [13].

#### 2.1.3. Bovine Viral Diarrhoea Virus (BVDV) Characteristics

Bovine viral diarrhoea virus (BVDV) is an enveloped positive single-stranded (ss) RNA virus, classified within the family Flaviviridae together with other pestiviruses including classical swine fever virus and border disease virus [14]. BVDV occurs as two distinct genotypes (genotype-1 and -2) [15]. BVDV is also classified as non-cytopathic (NCP) or cytopathic (CP) strains, of which the latter induces apoptotic cell death [16]. Bovine viral diarrhoea (BVD) is an OIE-listed disease of cattle with a worldwide prevalence and is well known for its impact on cattle health and production, leading to significant economic consequences by either direct losses or by costs of control and eradication programs [1,17,18]. The spread of BVDV in cattle populations is primarily caused by persistently infected (PI) animals, originally infected as fetuses in early gestation, after acute infection of the pregnant dam [19]. Besides cattle, other domestic species like pigs, goats and sheep can also become infected with BVDV causing reproductive disease [20,21,22,23].

#### 2.1.4. BVDV Intestinal Viral Load

BVDV was found in intestines in relative high titers up to. 7.3 log_10_ TCID_50_/g in the ileum at 6 dpi, while the highest blood titers at 7 dpi were 1.8 log_10_ TCID_50_/mL [24]. Maximum titers in the ileum and colon of 4 and 3.5 log_10_ were described at 10 days post-infection (pi), respectively. The virus was also present in the blood samples, but no titers were determined [25]. The highest relative expression levels of BVDV were found in ileum by qPCR at day 9 pi, while at the same time maximum expression levels in the blood were about 3.5 times lower [26]. Because, in general, the blood titers were considerably lower than the intestinal titers, it is most likely that replication of BVDV takes place in the intestines.

#### 2.1.5. Classical Swine Fever Virus (CSFV) Characteristics

Classical swine fever virus (CSFV) belongs to the same family as BVDV and is an enveloped positive single-stranded RNA virus [27,28]. Classical swine fever (CSF) is a serious, contagious OIE listed disease in pigs and wild boar, causing major damage to pig production worldwide [1,28,29]. CSF is an exotic disease in most developed countries whereas in other countries, e.g., in Asia, South-America and parts of Africa the virus is endemically present [30]. The “classical” form of CSF can be divided into acute form, resulting in high temperatures, diarrhoea and rapid onset of death (within 20 days) and chronic form, where apparent recovery from disease results in a chronic infection leading to death with infected animals surviving for as long as 100 days [31]. Vaccination with live-attenuated vaccines is mainly performed in endemic countries, while stamping out possibly in combination with emergency vaccination with marker vaccines is still the current policy when outbreaks occur in disease-free countries [28].

#### 2.1.6. CSFV Intestinal Viral Loads

CSFV was found in intestines in relative high titers up to 6.8 log_10_ TCID_50_/g in the ileum at 25 dpi, while on the same day the blood titers were 4.0 log_10_ [32]. High viral loads were also found by qPCR in the ileocecal valve after euthanasia or dead within a time period of 22 dpi [33]. Recently, pigs were experimentally infected with CSFV at slaughter age and euthanized at 10 dpi at the peak of viremia (4.4 log_10_ TCID_50_-eq/mL), after necropsy mean viral loads in the jejunum and colon of 3.7 ± 0.7 log_10_ TCID_50_/g were detected [11]. Virus detection and quantification by real-time RT-PCR showed maximum levels of CSFV in the small intestines around day 8 pi [34,35]. This timeframe was confirmed by antigen-ELISA results [36]. In bladder tissue, CSFV was present at lower levels than in intestines [34,36]. In blood, CSFV detection varied from equal to slightly higher levels compared to the levels found in the intestine samples, suggesting that viremia contributes to the viral load in the intestines [11,32,33,34,35]. 

#### 2.1.7. Foot and Mouth Disease Virus (FMDV)

Foot and mouth disease virus (FMDV) is a non-enveloped positive single-stranded RNA virus, classified within the Aphthovirus genus as a member of the Picornaviridae family [37]. Important livestock hosts include cattle, pigs, sheep and goats, but in general, FMDV affects all cloven-hoofed animals. The disease is characterized by the appearance of vesicular lesions in, and around, the mouth and on the feet [38]. There are seven major viral serotypes: O, A, C, SAT 1, SAT 2, SAT 3 and Asia 1 [39]. Serotype O is the most common serotype worldwide. It is responsible for a pan-Asian epidemic that began in 1990 and has affected many countries throughout the world until now [40]. Several serotype-based vaccines are commercially available, however, the development of novel FMDV vaccines to be used in endemic regions is still needed [41].

#### 2.1.8. FMDV Intestinal Viral Load

For FMDV, the efficacy of standard salt (NaCl) preservation to alternative P-salt treatment of natural casings derived from experimentally infected pigs, sheep and cattle at storage temperatures of 4 °C and 20 °C was investigated, and initial virus titers in the large and small intestines ranged from negative to maximum 3.0 log_10_ pfu/mL, while in cattle sera titers up to 3 log_10_ TCID_50_/mL were found [42,43]. When using PCR, FMDV was found in Peyer’s patches and serum in viral loads up to 4.32 and 6.27 log_10_ RNA copies/g at 2 dpi, respectively [44]. Similar PCR results were found and both studies indicate that titers were the result of the transportation of virus through viremic blood [45]. In cattle, no FMDV virus was found present in the epithelium of the urinary bladder [46]. 

#### 2.1.9. Hepatitis E Virus (HEV)

Hepatitis E virus (HEV) is a positive, single-stranded RNA virus without an envelope. HEV belongs to the genus Orthohepevirus in the family Hepeviridae. Swine HEV, which is closely related to human HEV, was discovered in the United States in 1997 and since then has been reported worldwide [47]. Two swine genotypes, HEV-3, endemic in Europa, America and Asia and HEV-4, mostly endemic in Asia, are known as zoonotic viruses capable of infecting humans by foodborne HEV transmission through the consumption of raw or undercooked pork [48]). In humans, HEV can cause besides hepatitis, neurological and renal diseases, while HEV infections in pigs usually are asymptomatic [49]. Hepatitis infections in pigs can therefore only be established by monitoring or surveillance programs. Currently, no commercial vaccines against HEV for either humans (except in China) or pigs are available [49,50]. 

#### 2.1.10. HEV Intestinal Viral Load

HEV was found present in the duodenum, jejunum, ileum, colon, caecum at copy numbers lower than 1000 RNA copies/g by PCR while blood titers were not determined [51]. Recently, a mean virus concentration of 3.3 × 10^3^ genome copies/g was found in the intestine of 69 wild boar in central Italy [52]. 

#### 2.1.11. Porcine Epidemic Diarrhoea Virus (PEDV)

Porcine epidemic diarrhoea virus (PEDV) is an enveloped RNA virus with a single-stranded positive-sense genome. It belongs to the family Coronaviridae which, together with the Arteriviridae family, constitutes the order Nidovirales [53]. The two genotypes of PEDV (S-INDEL and non-S INDEL) mainly affect neonatal piglets by acute diarrhea and/or vomiting, dehydration and high mortality, while infection of older animals results in minor or asymptomatic clinical symptoms. The non-S INDEL genotype is more virulent than the S-INDEL genotype and suddenly emerged in the PED-free United States in 2013, causing during a one-year epidemic period the loss of 8 million piglets. Nowadays, PED is considered as an emerging and re-emerging global enteric disease causing major economic losses in the swine industry worldwide [54,55,56]. An important strategy to control epidemic PED is to vaccinate pregnant sows to reduce the mortality rate of suckling piglets [57].

#### 2.1.12. PEDV Intestinal Viral Load

PEDV was present in intestine homogenates in a concentration of 2 to 6 log_10_ TCID_50_/mL, whereas blood titers were not determined [58,59].

#### 2.1.13. Peste des Petits Ruminants Virus (PPRV)

Peste des petits ruminants virus (PPRV) is an enveloped, negative-sense, single-stranded RNA virus with a single serotype, belonging to the genus Morbillivirus in the family Paramyxoviridae. Peste des petits ruminants (PPR) is an acute contagious disease, affecting mainly sheep and goats and occasionally wild small ruminants. Young animals (goats more than sheep) are the most severely affected, showing diarrhea and clear nasal discharge [60]. PPR occurs in Africa except for Southern Africa, in the Arabian Peninsula, throughout most of the Near East and Middle East, and in Central and South-East Asia. Several homologous cell culture-attenuated PPR vaccine strains are commercially available, providing strong immune responses [61,62]. In 2017 the FAO started the PPR global eradication plan (PPR-GEP) to eradicate PPR globally, similar to Rinderpest [63].

#### 2.1.14. PPRV Intestinal Viral Load

PPRV was found in intestines in relative high PCR titers up to 7.6 log_10_ RNA copies/g in the caecum at 8 dpi and in blood, PCR titers ranged between 0 and 2 log_10_ RNA copies/mL [64]. The most infected part of the intestine seems to be the caecum of sheep and goats where the highest level of genome copies was found. Besides in the caecum, viral RNA loads were also found in ileum, jejunum and colon. Because the RNA copy numbers in blood were lower than the intestinal titers it is most likely that PPRV may replicate in the intestine [65,66]. 

#### 2.1.15. Porcine Reproductive and Respiratory Syndrome Virus (PRRSV)

Porcine reproductive and respiratory syndrome virus (PRRSV) is an enveloped single-stranded positive-sense RNA virus and belongs to the same order Nidovirales as PEDV and TGEV, but is a member of the Arteriviridae family, genus rodartevirus [67]. There are two genetically and antigenically distinct genotypes, type 1 and type 2, originating from Europa (The Netherlands) and North-America, respectively. PRRSV causes acute reproductive failure in sows and mild to severe respiratory problems in new born and young pigs, which are the only known natural host [68,69]. Secondary infections by opportunistic pathogens may occur due to the immunosuppressive nature of PRRSV, resulting in more severe and chronic diseases. This immunosuppressive nature and the genetically/antigenically heterogenous genotypes of PRRSV are making vaccination and other control strategies a great challenge throughout the world [70].

#### 2.1.16. PRRSV Intestinal Viral Load 

PRRSV was detected in the intestines by immunohistochemistry (IHC) and in situ hybridization (ISH), however, no publications describing viral loads were found [71,72,73,74,75,76,77]. 

#### 2.1.17. Pseudorabies Virus (PrV)

Pseudorabies virus (PrV), also known as Aujeszky’s disease virus is an enveloped double-stranded DNA virus belonging to the family of the Herpesviridae, subfamily Alphaherpesvirinae, genus Varicellovirus. PrV can infect cattle, sheep, cats, dogs and rats causing fatal disease. Pigs, however, are able to survive and are therefore considered to be the only natural host. New born and nursing pigs are most severely affected showing mortality rates of 100% and 50%, respectively. Respiratory and neurological signs (mad itch) in weaned pigs and abortions in pregnant sows are hallmarks of this disease [78,79,80]. Eradication of PrV in large parts of the world was feasible due to efficacious marker vaccines in combination with accurate differential diagnostic tools [81]. 

#### 2.1.18. PrV Intestinal Viral Load

PrV was not detected in the intestines of infected pigs [82,83].

#### 2.1.19. Rinderpest Virus (RPV)

Rinderpest virus (RPV) is like PPRV, an enveloped negative-sense single-stranded RNA virus and member of the genus Morbillivirus, closely related to the measles and canine distemper viruses. Rinderpest (RP) was an infectious viral disease of cattle, domestic buffalo, and many other species of even-toed ungulates, including buffaloes, large antelope and deer, giraffes, wildebeests, and warthogs with high morbidity and mortality rates caused by oral and gastrointestinal ulcerations. In May 2011, the OIE declared that the disease was eradicated, making RP only the second disease in history to be fully wiped out, following smallpox [84,85]. Although RP was eradicated and additional measures of destructing worldwide laboratory virus stocks and live-vaccines were taken, the risk of re-emerging should not be considered negligible, therefore this virus was added to this literature search [86].

#### 2.1.20. RPV Intestinal Viral Load

RPV was found in intestines in relatively high titers (max. 6 log_10_ in the ileum at 10 dpi), while maximum blood titers were 2.6 log_10_ TCID_50_/mL [87]. Thus, virus replication in the intestines is most likely. Indirectly, this hypothesis was confirmed by the lack of pathogenicity of an attenuated RPV, primarily due to its failure to proliferate in the mucosae of the gastro-intestinal and respiratory tracts [88].

#### 2.1.21. Swine Vesicular Disease Virus (SVDV)

Swine vesicular disease virus (SVDV) is a non-enveloped positive single-stranded RNA virus and classified as an enterovirus within the family of Picornaviridae. Swine vesicular disease (SVD) is a contagious viral disease of pigs. It causes vesicular lesions in the mouth, on snout and/or feet and mucosa, indistinguishable from those observed of FMD [89,90]. Outbreaks were reported in Italy and Taiwan until the year 2000 and 2014, respectively. Currently, no outbreaks are reported worldwide because the disease is no longer listed as a notifiable disease, due to its mild nature and easy diagnosis by differential laboratory tests [91,92,93].

#### 2.1.22. SVDV Intestinal Viral Load

SVDV was found in the intestine at a maximum titer of 5.5 log_10_ TCID_50_/_mL_ in the ileum at 3 dpi, and in blood with a maximum titer of 4.6 log_10_ TCID_50_/_mL_ [94]. These results were confirmed by two studies showing similar titers in intestines and blood samples [95,96].

#### 2.1.23. Transmissible Gastroenteritis Virus (TGEV)

Transmissible gastroenteritis virus (TGEV) is, like PEDV, an enveloped virus with a positive single-stranded RNA genome that belongs to the genus Alphacoronavirus of the Coronaviridae family within the Nidovirales. The host is the pig and it leads to a mortality of nearly 100% in suckling pigs due to the replication of TGEV in the villous epithelial cells of the small intestine and lung [97]. Occurrences of TGEV have become more sporadic. The disease is still reported on an occasional basis from parts of Europe, North America and Asia [98,99]. Recently, an epidemic field strain TGEV (JS2012) was isolated from a pig farm in China and characterized as a novel natural recombinant virulent strain [100]. In the US, modified live and inactivated vaccines against TGEV are available, however, due to the declining prevalence, the need for vaccination is also declining [101]. 

#### 2.1.24. TGEV Intestinal Viral Load

TGEV was found in intestines in relative high titers (max. 7.5 log_10_ pfu/g at 4 dpi), however viral loads in blood samples were not determined [102]. This was confirmed by the presence of high levels of viral RNA (7–8 log_10_ RNA copies/g) in jejunum and ileum on the same day of post-infection [103]. Replication of a wild-type TGEV strain was also shown in the guts of piglets after intragastrical inoculation [104]. 

#### 2.1.25. Vesicular Exanthema of Swine Virus (VESV)

Vesicular exanthema of swine virus (VESV) is a non-enveloped, positive-sense, single-stranded RNA virus belonging to the genus Vesivirus in the family Caliciviridae [105]. VESV is clinically indistinguishable from FMDV, and in pigs, vesicles are formed on the snout, oral mucosa, soles of the feet, coronary bands and between the toes. VESV induced disease is highly infectious but rarely causes deaths. VESV first emerged in swine in California in 1932 and its origin was traced to feeding meat from sea mammals to pigs. VESV was eradicated from California in 1959. However, VES-like caliciviruses are likely widespread near the North American Pacific Ocean and occasionally appear in domesticated and captive wildlife in the Western United States, therefore VESV could still remain a threat to the United States swine industry. There is no vaccine against VESV available, the development of a vaccine is complicated due to the presence of multiple serotypes [106].

#### 2.1.26. VESV Intestinal Viral Load 

VESV was found in intestines in relative low titers (max. 1.8 log_10_ pfu/g in the colon at 4 dpi), blood titers were not determined [107]. 

#### 2.1.27. Vesicular Stomatitis Virus (VSV)

Vesicular stomatitis virus (VSV) is an enveloped, negative-sense RNA virus and belongs to the genus Vesiculovirus of the family Rhabdoviridae [108]. VSV is an arthropod-borne virus that primarily affects rodents, cattle, swine and horses, but can also infect humans and other species [109]. The clinical presentation, including vesicular lesions, is quite identical to FMDV. The virus is zoonotic and leads to a non-fatal flu-like illness in infected humans [108]. The disease occurs mainly in Central and South America, occasionally in the USA and rarely in Canada. No commercial vaccines are available [110].

#### 2.1.28. VSV Intestinal Viral Load

VSV was found negative in intestines and no viremia was detected [111].

Summarizing, published quantitative data on virus titers in intestines were found for all 14 viruses. The methodology in these publications was highly diverse on all aspects of research (e.g., animal species, ages, viruses used, tests used, quantification of virus), therefore comparison was only possible by a very general interpretation of results (Table 2). 

### 2.2. Q2 Inactivation Kinetics

From the 14 viruses used to search for the viral loads (Q1) in intestines and/or natural casings the following three viruses were selected for Q2: CSFV, BVDV and PPRV. The latter two viruses were selected because of their high viral loads in intestines in combination with low viremia titers indicating that the amount of virus present in the intestines is probably due to the replication of the virus in the intestinal wall. CSFV was selected as a “golden standard” because inactivation of this virus was previously determined in the 3D collagen matrix inactivation model [112]. 

The search concerning the inactivation kinetics of BVDV, CSFV and PPRV in the intestines, meat or food resulted in 8, 18 and 3 publications, respectively (Table 3).

#### 2.2.1. Inactivation of BVDV

No literature was found describing the inactivation of BVDV in intestines or casings, therefore it was impossible to determine D-values for processed natural casings or intestines. To get an impression about the inactivation kinetics of BVDV in other matrices, data were extracted from literature and, if possible, D-values were determined providing a starting point for future studies concerning natural casings. D-values of BVDV could be determined from the data provided in seven out of eight publications (Tables S15 and S16). Results were categorized into the following subgroups: processed meat, virus suspension, spiked virus and surface dried virus and D-values were determined by regression analysis of the extracted published data (Table S15). Because collagen is the main substance in casings, muscle and meat products are the most related matrices and were therefore added to the same group of processed meat. A virus suspension is a virus from a tissue/matrix that was liquefied by homogenizing in the medium. Spiked virus means that virus was added to a matrix, e.g., an immunoglobin solution or tissue.

Because BVDV in serum showed no decrease in titer for at least 5 days at 22 °C, no D-values from this publication could be calculated [113]. Muscle tissue from six BVDV persistently infected cattle was harvested, refrigerated, frozen and heated to various internal temperatures, only a D-value of 287.8 days could be determined by regression from the refrigerated data [114]. The thermostability study of BVDV suspensions in medium at a pH of 7, resulted in half-life values of 25 and 6 h at 21 °C and 37 °C, respectively [115]. These half-life values were converted to D-values: 2.91 (21 °C) and 0.97 (37 °C) days. BVDV spiked in medium at 5 °C, 20 °C, 35 °C, 40 °C, 45 °C, 50 °C and 55 °C was studied as a control to determine possible risk of spreading the disease by distributing slurry directly or after heat treatment on the farm land [116]. D-values were determined by regression, ranging from 15.72 days (20 °C) to 8.7 (55 °C) minutes for medium and 11.95 days (20 °C) to 1.0 (50 °C) minutes for slurry. The survival of surface-dried BVDV, suspended in either medium or plasma before drying on stainless steel, at room temperature was investigated for a period of 28 days, resulting in D-values by regression of 4.67 and 5.07 days, respectively [117]. Inactivation of surface-dried BVDV on stainless steel after 1 or 2 h at 40 °C, 75 °C, 85 °C and 95 °C was investigated, resulting in a D-value of 0.21 days at 40°C after regression [118]. The ability of the manufacturing process to inactivate/remove viruses and prions from a 10% liquid human intravenous immunoglobulin was investigated. An immunoglobin solution spiked with 5.4 log_10_ BVDV showed a log_10_ reduction of 0.0–0.4 when incubated at 2–8 °C (pH 7) for a period of 20 days [119]. From these data, a D-value of 50 days was determined by regression analysis. Animal feed ingredients spiked with BVDV, serving as a surrogate for CSFV, was incubated for 37 days in environmental chambers mimicking a trans-pacific transport. No viable BVD virus could be detected on all ingredients although all ingredients were spiked with 100 µL containing 5 log_10_ TCID_50_ and also this virus suspension, which was not added to any ingredient turned out negative at the end of the incubation period at 4–10 °C [120]. Regression analysis of the control sample resulted in a D-value of 7.4 days at this temperature range. 

#### 2.2.2. Inactivation of PPRV

No publications were found concerning inactivation in PPRV in intestines/casings, however, D-values could be calculated from data in three publications, which all investigated the thermostability of PPRV live attenuated vaccines (Tables S16 and S17). An alternative way of vaccine dehydration without the lyophilization step of sublimation from ice in the presence of disaccharide trehalose to protect the virus during desiccation was investigated [134]. From two studies, D-values were calculated from half-life values of live attenuated PPRV vaccines which were diluted in Weybridge medium without trehalose. Both studies showed comparable D-values at 4 °C and 37 °C [135,136]. The D-values obtained from these publications ranged between 179.5 and 73.1 days at 4°C and from 2.9 to 0.2 days at 45 °C.

#### 2.2.3. Inactivation of CSFV

From 16 out of 18 CSFV inactivation publications, D-values could be retrieved from the actual publication publications (3) or converted from published half-life data (3) or by regression analysis or from the data described (10) as indicated in Tables S18 and S19). Data were categorized into the following subgroups: intestines, blood, lymphatic tissue, tissues/processed meat, virus suspension, spiked virus and excretions (Table S19). A study investigated intestines from experimentally infected CSFV (virulent American strain) pigs after processing into natural casings by three different methods. After storage at 4 °C, these casings were subsequently fed to susceptible pigs to determine the infectivity of the remaining CSFV in these casings. The results showed that salt-treated casings handled at room temperature during processing were still infectious after 86 days, while casings incubated at 43 °C for 1 or 4 h during processing were no longer infectious after 17 and 0 days, respectively [121]. In another study, the intestines of two pigs infected with CSFV strain NADL (nowadays classified as a BVDV strain) were processed into casings, put in a saturated brine solution, and stored at 4 °C. After 147 days of storage, these casings were inoculated (route unknown) into two pigs. Both animals developed clinical symptoms and died, showing survival and infectiousness of CSFV in these casings even after this long storage period [10]. Supplementing salt with additives phosphate (P-salt) or citrate (C-salt) and storage at 4 °C and 20 °C, showed positive CSFV results by virus isolation on PK15 cells after 15 days of storage at both temperatures. At 30 days of storage, only the 4 °C casings stored in C-salt were found positive and were able to infect susceptible pigs after oral uptake. P-salt treated casings were found negative at both temperatures [122]. Unfortunately, in all three studies, the amount of virus in the intestines was not determined, making the calculation of D-values impossible.

Recently, D-values of CSFV infected intestines, processed with either saturated NaCl or phosphate-supplemented salt for 60 days at 4 °C, 12 °C, 20 °C and 25 °C were published, and ranged for NaCl from 18.3 (4°C) to 3.4 days (20°C) [11]. Previously, similar D-values were determined under comparable conditions by the use of a 3D collagen model during a period of 30 days [112]. A study on thermal inactivation of CSFV infected serum published D-values ranging from 219.92 h at 25 °C to 0.53 min at 68 °C [123]. Another study investigated thermal inactivation of CSFV with a titer of 5 log_10_ TCID_50_/mL in defibrinated swine blood. CSFV was inactivated when heated to 69 °C, 68 °C and 66 °C for 30, 45 and 60 min, respectively. Regression analysis indicates D-values ranged from twelve (66 °C) to six (69°C) minutes [124]. After intramuscular inoculating grounded lymph nodes with a CSFV titer of 1.9 log_10_ pfu/g, followed by heating at 69 °C for 15 min, none of the two pigs showed signs of CSFV, resulting in a D-value of 7.3 min [10]. The decay at room temperature of infectious CSFV in organs from infected pigs was expressed in half-life values in days, which were converted into D-values of 0.9, 0.7 and 0.96 days for spleen, tonsil and lymph node, respectively [125]. Thermal inactivation of lymph nodes derived from pigs infected with CSFV was investigated at several temperatures, and the published D-values ranged from 15.5 days (4 °C) to 2.4 min (56 °C) [123]. CSFV suspensions containing organ tissues (spleen, lymph nodes, kidney, muscle, tonsil, bone marrow, viscera) were inactivated at temperatures ranging from 80 °C to 160 °C. Viable virus was still found after 60 min at 90 °C while CSFV was inactivated after 5 min at 16 0°C. Regression analysis of the data resulted in D-values ranging from 0.1 (110 °C) to 0.01 (160 °C) minutes [126]. Inactivation of CSFV infected fat and muscle resulted in D-values ranging from 10.57 days and 39.2 days at 4 °C and 1.15 and 0.41 min at 68 °C, respectively [123]. At several timepoints during the curing process of shoulder hams derived from CSFV infected pigs in the USA and Italy, muscle, bone and fat samples were taken and the infectivity of these samples was examined by virus titration and in vivo experiments [127]. Because no initial titers of the Italian hams were mentioned, the regression analysis was performed on data from days 32 until 189. Since the major curing period (90 days) took place at temperatures between 2.5–6.4 °C as described in a previous publication of the same author, regression analysis was set at 4 °C and D-values ranged from 73 to 25 days [137]. Survival of CSFV in Iberian ham, shoulder, loin and Serrano ham derived from infected pigs, showed no detection of the infectious virus after completion of the curing period, with D-values determined by regression ranging from 46.7 to 10.6 days [128]. Thermostability of CSFV suspensions in medium at a pH of 7, resulted in mean half-life values of 50 and 7 h and converted to D-values of 6.92 days and 0.97 days at 21 °C and 37 °C, respectively [115]. Furthermore, thermal inactivation of a CSFV vaccine strain and several local Indian isolates, adapted to the PK-15 cell-line, was determined at 4 °C, 27 °C and 60 °C, D-values obtained by regression ranged between 22.9 days (4 °C) to 2.19 min (60 °C) [129]. CSFV spiked in either slurry or cell-culture medium was studied at 55 °C, 60 °C, 65 °C and 70 °C. Regression of these data showed similar D-values for either medium or slurry ranging from 1.29 to 0.4 min at 55 °C and 70 °C, respectively [130]. A similar study with CSFV spiked in either slurry or medium at lower temperatures (5 °C, 20 °C, 35 °C, 40 °C and 50 °C) was performed to determine to possible risk of spreading the disease by distributing slurry directly or after heat treatment on the farm land [116]. D-values obtained by regression ranged between 18.5 (slurry) and 55.5 days (medium) at 5 °C to 43 (slurry) and 28 min (medium) at 40 °C. Survival of surface-dried CSFV, suspended in either medium or feces before drying on stainless steel, at ambient temperature was investigated for a period of 5 h, regression analysis showed D-values of 0.51 (medium) and 0.05 days (feces) [131]. The stability of a bread-based lyophilized C-strain CSF virus vaccine as an oral vaccine in pigs was tested at 4 °C, 25 °C and 37 °C for 7 days. At 4 °C, the vaccine with a titer of 3.7 log_10_ TCID_50_/_mL_ remained stable for 7 months, while for 25 °C and 37 °C D-values could be calculated by regression resulting in 1.35 and 0.97 days, respectively [132]. Survival of two CSFV strains at various temperatures in feces and urine derived from experimentally infected pigs was examined and expressed as half-life values, which after converting showed D-values ranging between 11.6 (feces) and 9.4 days (urine) at 5 °C to 0.3 (feces) and 0.2 days (urine) at 30 °C [133].

Summarizing the inactivation results, no publications about inactivation kinetics in intestines were available for BVDV and PPRV, while for CSFV only a limited amount of publications was available. Therefore, the majority of D-values were obtained or determined to achieve an impression about the inactivation kinetics in other matrices. All three viruses showed thermal dependent inactivation. 

#### 2.2.4. Statistical Analysis

**Figure 1:**
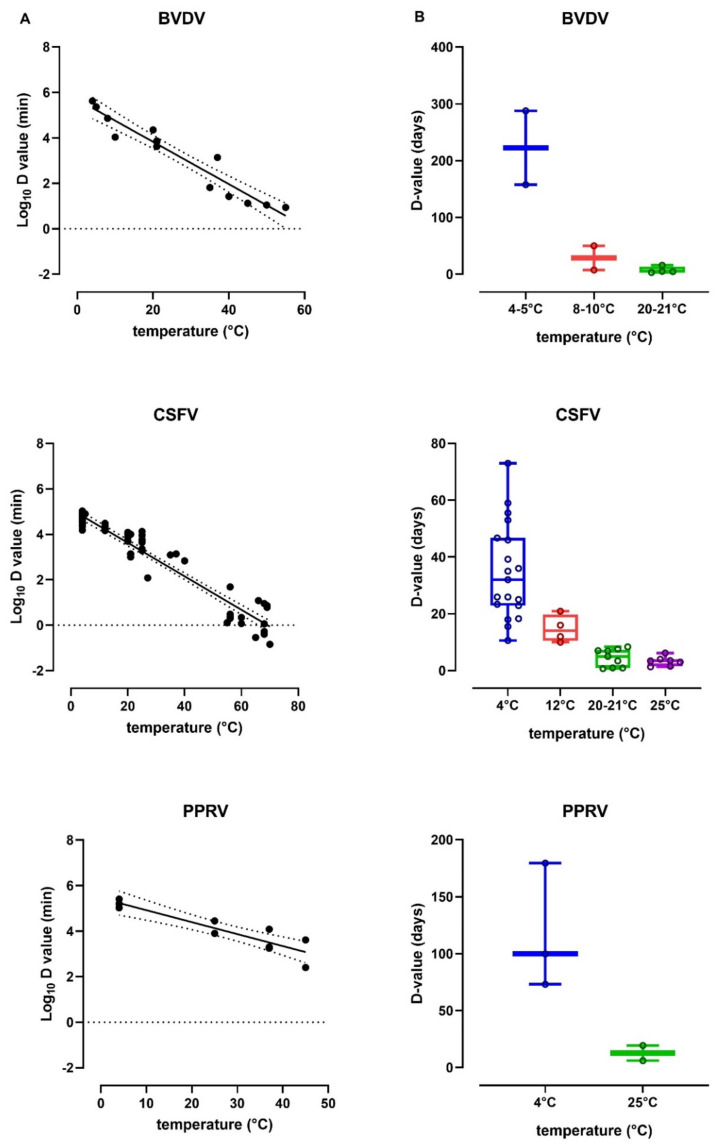
(**A**). Regression analysis of selected log10 D-values in minutes against temperatures based on 6, 13 and 3 publications for BVDV, CSFV and PPRV, respectively, as indicated in Tables S16 and S19 in red. Dotted lines indicated 95% confidence intervals. (**B**). Box and whisker plots of calculated D-values per virus over a temperature range of 4 °C to 25 °C. Individual D-values are indicated by colored rings.

In order to approach intestine and/or casing tissues as close as possible, D-values obtained from slurry, excretion, secretion, bread-based and surface-dried studies were excluded, therefore regression analysis was performed on D-values obtained from 6, 13 and 3 publications of BVDV, CSFV and PPRV, respectively (Tables S15–S20). The selected D-values in days of each virus were log-transformed in log_10_ D-values in minutes which enabled plotting against temperature (Figure 1A). Subsequent regression analysis and back transformation made it possible to estimate D-values in days at temperatures important for the casing industry: 4 °C, 12 °C, 20 °C and 25 °C (Table S20). Additionally, selected non-transformed D-values at these temperatures were plotted in maximum and minimum box and whisker plots (Figure 1B). The descriptive statistics of these plots, including mean and SEM data are indicated in Table S21.

## 3. Discussion

The aim of Q1 was to perform a literature search using search engine CAB Abstracts to determine the viral load of 14 selected animal viruses in the intestines of the host animal.

For all 14 viruses, between zero and six relevant publications were found, which include quantitative data on virus titers in intestines. Quantitative data on virus distribution over anatomically distinct parts of the intestines are limited and available (at least to some extent) for ASFV, BVDV, CSFV, PPRV, RPV, SVDV and TGEV. With the exception of VSV, PrV and PRRSV, viral loads of all viruses were detected in tested parts of the intestines. Maximum titers were highest for ASFV, BVDV, CSFV, PPRV (qPCR only), RPV and TGEV. Maximum titers for these viruses reached > 6 log_10_ TCID_50_/g of tissue, therefore these viruses could be of major concern for the casing industry. However, quantitative data on a more detailed virus distribution in separate layers of the intestines (mucosa, submucosa, muscular layer, serosa) were not available, while the majority of the natural casings are made of the submucosa layer of the small intestine. The ileum comprises the part of the intestines that was most frequently tested for the presence of a virus. The amount of Peyer’s patches is in the ileum higher than in the other parts of the intestines. Because of their involvement in Bovine Spongiform Encephalopathy (BSE) the ileum section of the small intestine of cattle will not be processed to natural casings, while ileum sections of pigs and small ruminants are not removed [138,139]. Because the other intestinal parts were less investigated, it is difficult to conclude whether the risks of virus decontamination by excluding the ileum from the casing process will be diminished. High viral loads in intestines in combination with low viremia titers will most likely correlate with replication of the virus in the intestines, and may subsequently increase the chance of detectable virus remaining after casing production. However, virus replication may also lead to tissue damage at the site of replication, thus increasing the chance of post-mortem detection at the time of slaughter. 

The current SARS CoV2 pandemic has led to the evaluation of whether this virus is also relevant for the casing industry. Experimental studies with SARS CoV2 in farm animals have demonstrated that cattle and pigs were low susceptible and resistant to SARS CoV2, respectively [140,141,142]. Therefore the risk of spreading SARS CoV2 via porcine and bovine casings is estimated to be negligible. 

Due to the limited number of publications on intestinal viral loads, it is quite difficult to exactly define which viruses may be of threat for the casing industry, because the majority of these publications were not performed with this specific goal in mind. Besides viruses identified with the highest titers, other viruses might still be of concern. For example, PPRSV was detected in intestines in many publications but none of these publications investigated the viral loads. Additionally, the high diversity in methodology between publications, like animal species, ages, virus strains, inoculation dose, time of euthanasia, experimental set-up and virus titration methods could have a major impact on the amount of virus detected in the intestines. As a result, the amount of virus present in the intestines could be either under- or overestimated, which in both cases could have major consequences for the casing industry by possible trade limitations.

Three viruses found in the intestines with high viral loads, CSFV, BVDV and PPRV, were selected for Q2 (inactivation) of the literature search. This search resulted in no findings of inactivation data (titers, D-values) regarding these viruses in sausage casings, and therefore relevant data to determine quantitative inactivation parameters in sausage casings is non-existent. For CSFV, a reasonable number of inactivation publications were suitable for statistical analysis, while for BVDV and PPRV much less publications were available. Nonetheless, the obtained data can serve as informative guidance for future inactivation studies specific to the casing industry. Besides differences in virus strains, detection methods, spiking versus infected tissues, animal studies, matrices and storage/incubation methods, extracting data for D-value calculation sometimes required additional assumptions concerning graphical data and/or initial titers. The height of the calculated D-values will be influenced by all these different factors and may lead to possible under- or overestimations. For example, D-values of PPRV will probably be overestimated, because studies were limited to vaccine inactivation experiments in combination with stabilizing ingredients. Another example is the determination of the D-value of BVDV from a publication where the initial titer (day 0) is lower than titers at days 2, 7, 14 and 21 during the inactivation [114]. Using all data (including day 0) resulted in a D-value of 288 days at refrigeration temperature (Table S16), while a D-value of 51 days was found when the initial titer was excluded from regression analysis (Table S15). 

In some studies, different individual responses to virus infection were observed, e.g., feeding CSFV infected tissue (casings or ham) to susceptible pigs, showed some inconsistencies for both types of tissues, which might be caused by different initial viral loads of individual infected pigs [121]. Another study describes a single animal with sufficient CSFV titers in muscle tissues due to an underlying infection [123]. Recently, one out of two pigs infected with CSFV at the age of 6 months showed insufficient titers in both small and large intestines [11]. These additional findings may form important parameters for risk assessment modeling studies. Clear scientific data about the inactivation kinetics of viruses by the standard salt treatment are very important for the casing industry. A 3D collagen matrix model was developed, mimicking the natural casing and ensuring maximum virus titers to be tested in combination with the standard salt treatment [112]. This in vitro test system enables the determination of D-values of each virus, avoiding the described limitations when performing animal experiments. Although saturated salt is still the standard procedure for preserving natural casings, other preservatives or salt-additives can be also tested in this in vitro system to determine possible faster inactivation kinetics. 

Some publications about viral load and inactivation in languages other than English or German could have been missed for this SR. No relevant German publications were found in both searches. Because English is the common language for publishing, the chance of a possible bias in this SR will be quite low.

In conclusion, this SR shows that major gaps exist when it comes to viral loads and inactivation of viruses in sausage casings or even the intestines from which the sausage casings are produced. A systematic research approach will be needed to fill these knowledge gaps and develop efficacious and efficient protocols for the inactivation of relevant viruses in relation to possible trade barriers.

## 4. Materials and Methods 

### 4.1. Search Questions 

Q1. What are the virus loads of 14 selected viruses (Table 1) in intestines?

Q2. What are the inactivation kinetics (D-values) of three viruses with high intestinal viral loads selected from Q1?

### 4.2. Search Strategy and Methodology

This SR basically followed the Preferred Reporting Items for Systematic Reviews and Meta-Analyses (PRISMA) guidelines, including quality assessment and transparency [143]. The search consisted of 4 steps: Literature search (1), Screening and quality assessment (2), data extraction (3) and data analysis and summation (4). All steps were executed by one reviewer. Prior to the start, a protocol was prepared by defining variables and outcomes (Table S22). The search for relevant publications was performed using the CAB Abstracts database provided by the Wageningen University and Research Library (Wageningen UR, The Netherlands). Keywords were defined and used to search in the title, abstract and keywords. The search language was in English. Searching in additional databases, Scopus (Elsevier, The Netherlands) and Google Scholar, was performed when CAB Abstracts resulted in a limited number of positive hits, but no additional results were found. The keywords, as indicated in Table 1 and Table 3, were entered in CAB Abstracts, starting with the name of the virus combined with the other keywords by using OR forming a final search string. CAB Abstracts searched automatically both the single and plural form of a keyword without adding any special code. CAB Abstracts searched on the entire phrase instead of separate words, e.g., African Swine Fever. Besides that, to increase the search power, the asterisk (*) was used the end of the root of a word to instruct the database to search for all forms of a word, e.g., pathog* retrieved pathogenicity or pathogenesis. The search included synonyms of virus names found with the thesaurus search tool of CAB abstracts. No time-limits were imposed on the publication date and the last search was performed on 18 May 2020. 

### 4.3. Data Extraction

After entering the keywords in CAB Abstracts, titles and abstracts were first screened for possible information about either Q1 or Q2. The aim of this first screening was to exclude non-relevant publications and select possible relevant publications. Titles and abstracts were screened for correct virus nomenclature, type of research and language, publications in a language different from English and German were omitted from selection. Additional criteria for Q1 were: publications describing viral loads, quantified by virus titration or qPCR of the subject virus in intestines, gastro-intestinal tract, ileum, jejunum, duodenum, colon or bladder. This search included primary research in either field or experimental studies on pathogenicity, infection, vaccine/challenge or inactivation studies, to gain as much information as possible. In the case of vaccination/challenge and inactivation studies, data were extracted from the non-vaccinated challenged control animals or initial titers before inactivation. The additional criteria for Q2 were: publications on either BVDV, PPRV and CSFV describing experimental studies about inactivation, survival, viability, stability and disinfection in intestines, casings or other matrices. Only studies that complied with all criteria of either Q1 or Q2 were included for a second screening. The most common reason for excluding were non-relevant studies in which the goal virus was not the main research subject. In case it was not clear whether a study met all criteria it was included for the second screening to avoid missing out on relevant data. The selected publications were imported in EndNote X9 and duplicates were removed (step 1). The full-text publications were retrieved through online availability or via the Library Servicedesk of Wageningen UR. These publications underwent a second screening assessing the quality of the published data (step 2), including only publications where the search question (Q1 or Q2) could be answered. For Q1, these criteria were: compliance to the Q1 first screening criteria and viral loads should be expressed as 50% tissue culture infective dose (TCID_50_), plaque-forming units (pfu), 50% Haem-absorbing doses (HAD_50_) or TCID_50_-equivalents by qPCR, genomic copies by PCR or Ag-ELISA. For Q2, the criteria were: compliance with the Q2 first screening criteria and inactivation data should be quantified by virus titration and expressed in TCID_50_ or pfu. Or publications should describe regression analysis resulting in D-values or half-lives. The main reason for excluding was the failure of describing intestinal viral loads (Q1) or inactivation kinetics (Q2). The in- and excluded publications and flowcharts of Q1, Q2, listed per virus, and the PRISMA checklist are indicated in S23–S36, S37–S39 and S40, respectively. Data from the relevant full-text publications were recorded and summarized in an Excel spreadsheet. For Q1, the following headings were used: reference, virus, virus strain, animal species, inoculation dose, sample type, number of samples, assay, experimental or field study, days post-inoculation, intestine, duodenum, jejunum, Peyer‘s patches, ileum, colon, caecum, bladder, blood and remarks. Publications describing viral loads by quantitative PCR (qPCR) or ELISA instead of virus titration were included in the Excel sheet with a search result (Tables S1–S14).

For Q2, the following headings were added to an excel sheet: type of research, inactivation time, inactivation temperature, pH, submucosal membrane, muscular layer, natural casing, viral load, inactivation method, half-life, D-value (time to reach a 1 log_10_ decrease of virus titer) as indicated in Tables S15, S17 and S18. In case D-values were not indicated in a publication, then if possible, data were extracted from graphs and/or tables to calculate D-values for each specific matrix by regression analysis in which the negative reciprocal of the slope of the trendline represents the D-value. Half-life values were converted to D-values by dividing Half-life with Log_10_(2). Titers expressed in plaque-forming units (pfu) were transformed into TCID_50_ by dividing the pfu titer with 0.7 [144]. 

For a correlation in temperature, the retrieved or calculated D-values in the different matrices were log-transformed and categorized (in minutes) in an Excel spreadsheet. These log_10_ D-values were plotted against the temperatures and regression analysis using Graphpad Prism version 8 enabled the interpolation and extrapolation of virus inactivity rates at different temperatures and D-values were estimated for relevant temperatures (4 °C, 12 °C, 20 °C and 25 °C) for BVDV, CSFV and PPRV. Besides this analysis, Graphpad Prism version 8 was used to perform descriptive statistics and box and whisker plots combined with individual data from non-transformed D-values of BVDV, CSFV and PPRV at relevant temperatures ranging from 4 °C to 25 °C. 

## Figures and Tables

**Table 1:** Results search question 1 (Q1).

String Keywords	Results String	Virus Keywords	Results Virus	Results Step 1	Step 1 Excl.	Results Step 2	Step 2 Excl.	Final
Pathogenes *	98403	African swine fever or ASFV or Warthog virus	3032	82	38	44	39	5
Intestin * or Gastrointestin * or digestive tract	272212	Bovine diarrhoea virus or Bovine viral diarrhea virus or Bovine viral diarrhoea virus or mucosal disease	7899	375	315	60	57	3
Ileum or Jejunum or duodenum or colon	65647	Classical swine fever virus or CSFV or HCV Hog cholera virus or swine fever virus	10533	392	342	50	44	6
Tissue tropism or tissue distribution	10937	Foot-and-mouth disease virus or FMD virus or FMDV or Foot and mouth disease virus	12449	184	170	14	9	5
Bladder or urinary bladder	18134	Hepatitis E virus or Hepatitis E	1961	74	64	10	8	2
Viraemia or Tit * or Viral load	202550	Porcine epidemic diarrhea virus or PEDV	1465	326	241	85	83	2
		Porcine respiratory and reproductive syndrome virus or PRRSV or SIRS or swine infertility and respiratory syndrome virus or Porcine Epidemic Abortion and Respiratory Syndrome or PEARS	36928	234	210	24	24	0
		Peste-des-petits-ruminants virus or pest of small ruminants virus or Peste des petits ruminants virus or PPRV	1571	68	49	19	16	3
		Pseudorabies virus or PRV or Aujeszky virus or Aujeszky’s disease virus or Aujeszky’s virus SuHV-1	7445	172	156	16	14	2
		Rinderpest virus or Rinderpest	4396	150	143	7	5	2
		Swine vesicular disease or SVD	1067	34	26	8	5	3
		Transmissible gastroenteritis virus or Porcine transmissible gastroenteritis virus or TGEV	2207	394	370	24	21	3
		Vesicular exanthema of swine virus or Swine vesicular exanthema virus or vesicular exanthema virus or VESV	141	6	4	2	1	1
		Vesicular stomatitis virus or VSV	2849	62	57	5	4	1

* was used the end of the root of a word to instruct the database to search for all forms of a word.

**Table 2:** Overview viral loads found in intestines, bladder and blood.

Virus	Intestines	Duodenum	Jejunum	Ileum	Caecum	Colon	Bladder	Blood	Source
ASFV	++++	++++	++++	++++	+++	++++	nd	+++++	[9,10,11,12,13]
BVDV	++++	nd	+++	++++	+++	+++	nd	+	[24,25,26]
CSFV	++++	(+++)	++++	++++	nd	+++	(++)	++++	[11,32,33,34,35,36]
FMDV	+	(+)	nd	nd	nd	+	-	++	[42,43,44,45,46]
HEV	(++)	nd	nd	nd	nd	nd	nd	nd	[51,52]
PEDV	+++	nd	nd	nd	nd	nd	nd	nd	[58,59]
PPRV	(++++)	(++++)	nd	(++++)	(++++)	(++++)	nd	(++)	[64,65,66]
PRRSV	nd	nd	nd	nd	nd	nd	nd	nd	-
PrV	-	nd	nd	-	nd	-	nd	nd	[82,83]
RPV	++++	nd	nd	++++	++++	+++	nd	++	[87,88]
SVDV	+++	+++	+++	+++	++	++	nd	+++	[94,95,96]
TGEV	++++	nd	++++	++++	nd	nd	nd	nd	[102,103,104]
VESV	+	nd	nd	nd	nd	+	nd	+	[107]
VSV	-	-	nd	-	nd	-	nd	-	[111]

-: No titer found. +: Titer <10^2^. ++: Titer 10^2^–10^4^. +++: Titer 10^4^–10^6^. ++++: Titer 10^6^–10^8^. +++++: Titer >10^8^. ( ): Titer PCR. nd: not determined.

**Table 3:** Results search question 2 (Q2).

String Keywords	Results String	Virus Keywords	Results Virus	Results Step 1	Step 1 Excl.	Results Step 2	Step 2 Excl.	Final	Source
Inactivat *	60748	Bovine viral diarrhea virus or Bovine diarrhea virus or Bovine diarrhoea virus or BVDV or BDV or mucosal disease	8524	2580	2553	27	19	8	[113,114,115,116,117,118,119,120]
surviv *	311829	classical swine fever virus or Hog cholera virus or CSFV or CSF	16667	8205	8136	69	51	18	[10,11,112,115,116,121,122,123,124,125,126,127,128,129,130,131,132,133]
decimal reduction valu * or D-valu *	2862	Peste des petits ruminants or PPRV	1626	453	438	15	12	3	[134,135,136]
therm * inactivat *	2515								
pH or pH stabil *	404282								
salin* or saline stabil *	161523								
Brine	9430								
Half-life	18643								
therm * stabil * or temperature	640766								
casing or sausage	8101								
Intestin *	209983								
meat or food	2259753								

* was used the end of the root of a word to instruct the database to search for all forms of a word.

## Data Availability

All data presented in this study are available in this manuscript and supplemental materials.

## Supplementary Materials

The following are available online at https://www.mdpi.com/2076-0817/10/2/173/s1, Supplementary 1: Q1 publications, Table S1: African swine fever virus (ASFV), Table S2: Bovine viral diarrhea virus (BVDV), Table S3: Classical swine fever virus (CSFV), Table S4: Foot and mouth disease virus (FMDV), Table S5: Hepatitis E virus (HEV), Table S6: Porcine epidemic diarrhea virus (PEDV), Table S7: Peste des petits ruminants virus (PPRV), Table S8: Porcine reproductive and respiratory syndrome virus (PRRSV), Table S9: Pseudorabies virus (PrV), Table S10: Rinderpest virus (RPV), Table S11: Swine vesicular disease virus (SVDV), Table S12: Transmissible gastroenteritis virus (TGEV), Table S13: Vesicular exanthema of swine virus (VESV), Table S14: Vesicular stomatitis virus (VSV), Supplementary 2: Q2 publications, Table S15: Bovine viral diarrhea virus (BVDV), Table S16: Calculated D-values per temperature from BVDV and PPRV Q2 publications, Table S17: Peste des petits ruminants virus (PPRV), Table S18: Classical swine fever virus (CSFV), Table S19: Calculated D-values per temperature from CSFV Q2 publications, Table S20. Regression analysis of selected log10 D-values in min as plotted in Figure 1A and backtransformed to D-values in days at 4 °C, 12 °C, 20 °C and 25 °C, Table S21: Descriptive statistics selected D-values (days) per virus at 4 °C to 25 °C as plotted in Figure 1B (Box and Whiskers), Table S22: Summary of the systematic review protocol, Supplementary 3: In/exclusion publications Q1 and Q2, Table S23: Q1 ASFV publications, Table S24: Q1 BVDV publications, Table S25: Q1 CSFV publications, Table S26: Q1 FMDV publications, Table S27: Q1 HEV publications, Table S28: Q1 PEDV publications, Table S29: Q1 PRRSV publications, Table S30: Q1 PPRV publications, Table S31: Q1 PrV publications, Table S32: Q1 RPV publications, Table S33: Q1 SVDV publications, Table S34: Q1 TGEV publications, Table S35: Q1 VESV publications, Table S36: Q1 VSV publications, Table S37: Q2 BVDV publications, Table S38: Q2 PPRV publications, Table S39: Q2 CSFV publications, Supplementary 4, Table S40: PRISMA Checklist.
